# Projected population exposure to dangerous heat stress around Lake Victoria under a high-end climate change scenario

**DOI:** 10.1088/1748-9326/ae05b1

**Published:** 2025-09-23

**Authors:** Delphine Ramon, Clare Heaviside, Oscar Brousse, Charles Simpson, Irene Amuron, Eddie Wasswa Jjemba, Jonas Van de Walle, Wim Thiery, Nicole P M van Lipzig

**Affiliations:** 1 Department of Architecture, Faculty of Engineering Science, KU Leuven, Leuven, Belgium; 2 Institute of Environmental Design and Engineering, Bartlett School of Environment Energy and Resources, University College London, London, United Kingdom; 3 Red Cross Red Crescent Climate Centre, The Hague, The Netherlands; 4 Department of Earth and Environmental Sciences, Faculty of Science, KU Leuven, Leuven, Belgium; 5 Department of Hydrology and Hydraulic engineering, Vrije Universiteit Brussel, Brussels, Belgium

**Keywords:** heat stress, population exposure, Lake Victoria, convection-permitting, regional climate modelling, climate change

## Abstract

Recent global temperature increases and extreme heat events have raised concerns about their impact on health, particularly in vulnerable regions like Africa. This study assesses future heat stress and population exposure in the Lake Victoria region under the high-emission SSP5-8.5 climate change scenario, using a convection-permitting climate model, heat stress indices (humidex and heat index), and high-resolution population projections under the high-emission SSP5-8.5 scenario, interpreted here as the high-end of the climate change signal. Results indicate a substantial increase in the duration of dangerous heat stress. By the end of the century, up to 122 million people, or around 44$\%$ of the population may experience dangerous heat stress for more than 5$\%$ of the time annually (i.e. ∼18 days), compared to 1$\%$ of the population or around 1 million people for the period 2005–2016. Up to 28$\%$ of the population (∼78 million people) would even experience dangerous heat for 15$\%$ of the time (i.e. ∼55 days). 66$\%$ of this increased population exposure can be attributed to the combined effect of increasing temperatures and total population in the region. High heat-risk areas include the northern and southern shores of Lake Victoria and urban areas. The study highlights the need to consider both climate and population dynamics when assessing heat stress, and underscores the urgency of adaptation in the Lake Victoria region.

## 1. Introduction

Recent climate changes have led to a substantial increase in global temperatures and in extreme heat (IPCC 2022),which have been correlated with higher rates of mortality and morbidity throughout the world (Liu *et al* 2017, Vicedo-Cabrera *et al* 2021, Chapman *et al* 2022, IPCC 2022, Lüthi *et al* 2023). Therefore, future increases in global temperatures are anticipated to exacerbate unfavourable health outcomes, including amplified heat stress (Liu *et al* 2017).

The African continent emerges as one of the most susceptible regions to climate change and heat stress (Russo *et al* 2016, IPCC 2022, Van de Walle *et al* 2022). Multiple studies have already projected an increased exposure to extreme heat, thereby accentuating the rise of harmful thermal conditions.

According to Thiery *et al* (2021), children born in 2020 in sub-Saharan Africa will encounter up to 57 times more heatwaves in their life than they would have without climate change. Globally, their counterparts are expected to encounter 41.4 times more heatwaves.

Liu *et al* (2017) projects a substantial increase in heatwave frequency in Africa, potentially mounting up to a 120-fold higher occurrence by 2071–2100 compared to 1971–2000. Asefi-Najafabady *et al* (2018) projects amplifications in exposure to heat stress (here the number of days with an apparent temperature exceeding 39 ^∘^C) ranging from 7 to 269 times the current exposure under representative concentration pathway (RCP) 8.5 across the Great Lakes, with highest exposures in Democratic Republic of the Congo and Uganda.

The region around Lake Victoria is home to more than 45 million inhabitants and is one of the most densely populated areas in Africa (The World Bank 2018). The region is also characterised by rapid population growth and urbanisation (Liu *et al* 2017, Asefi-Najafabady *et al* 2018, United Nations 2022). Most countries in this region will encounter a doubling in population by 2050 compared to 2022 (United Nations 2022).

Additionally, there is a lack of research on the impact of climate change, specifically regarding heat stress. This research gap is partly due to limited availability of meteorological and health data (Asefi-Najafabady *et al* 2018, Barbier *et al* 2018, Brousse *et al* 2020, Vicedo-Cabrera *et al* 2021, Chapman *et al* 2022, Van de Walle *et al* 2022). Moreover, it is a region with large heterogeneity induced by the presence of the lake and the orography, which cannot be captured by the coarse resolution of global climate models.

Recently, convection-permitting (km-scale) regional climate models have been applied to this region in the context of the CORDEX Flagship Pilot Study climate Extremes in the Lake VICtoria basin (van Lipzig *et al* 2023), as well as in a pseudo global warming experiment (Van de Walle *et al* 2021).

Specifically for heatwave related assessments, the research of Birch *et al* (2022) found a better representation of humid heatwaves at convection-permitting scale compared to model simulations with parametrised convection across the African domain. Further, the pseudo global warming experiment uses an ensemble mean climate change signal which is more robust compared to the signal from individual members (Van de Walle *et al* 2021). Such an approach is sensible in this region characterised by a large spread among different ensemble members (van Lipzig *et al* 2023).

The combination of an increasing population and extreme heat makes the region around Lake Victoria particularly susceptible to future heat stress. Liu *et al* (2017) found that the confluence of augmented heatwave occurrences and population expansion largely attributes to the increase in heat exposure for this region. Furthermore Asefi-Najafabady *et al* (2018) stress the need for high-resolution climate model projections due to the spatial heterogeneity in the magnitude of projected warming across this region. These high-resolution models offer hourly data, which is essential for studying heat stress.

This paper investigated the impacts of climate and population changes on heat exposure and heat stress in the region surrounding Lake Victoria. We analysed how dangerous heat stress could change under the ensemble mean climate change signal of the high-end SSP5-8.5 climate change scenario and how the local population could be affected by these changes. A high-end climate change scenario was chosen to define robust climate adaptation measures. The research is focused on three main questions: (1) To what extent will dangerous heat stress change due to climate change? (2) To what extent will heat exposure change for the population in the region? (3) Where are people exposed to dangerous heat stress localised? To answer these questions, we used a combination of convection-permitting climate modelling, heat stress metrics (i.e. humidex and heat index), and population density data.

By doing so, we gain a better understanding of the potential consequences of climate change and heat stress on the people living around Lake Victoria. This knowledge is crucial in developing strategies and interventions to mitigate the negative impacts of climate change and to promote long-term sustainability in the region. Advancing previous research on the topic, our analysis uses climate data generated at a 2.8 km grid spacing which is an added value compared to previous studies using CORDEX (12 km) or GCM (∼100 km) data, especially in this region of complex orography, presence of lakes and variable land use. In addition, changes in mesoscale circulation related to topography and temperature contrast of lake areas compared to land areas are accounted for. We achieve this by employing high-resolution (i.e. grid spaces of 2.8 km) regional climate projections using a pseudo-global warming approach. This approach proves crucial for enhancing the accuracy of climate representation. Moreover, an in-depth evaluation of top-of-the-atmosphere radiation for convection-permitting models (CPMs) compared to models with parametrised convection revealed a significant improvement in the diurnal cycle (van Lipzig *et al* 2023). This indicates that CPMs better represent the diurnal cycle of clouds, which is crucial for accurately assessing extreme daily temperatures and moisture, and therefore, for heat stress metrics.

Furthermore, our study combines exposure to heat stress, not only due to climate change but also due to population growth, in a consistent SSP-RCP-based way. This is particularly relevant because aligning climate data granularity with population density granularity is essential in understanding exposure to heat stress

## 2. Data and methodology

### 2.1. Regional climate model

**Figure 1.**
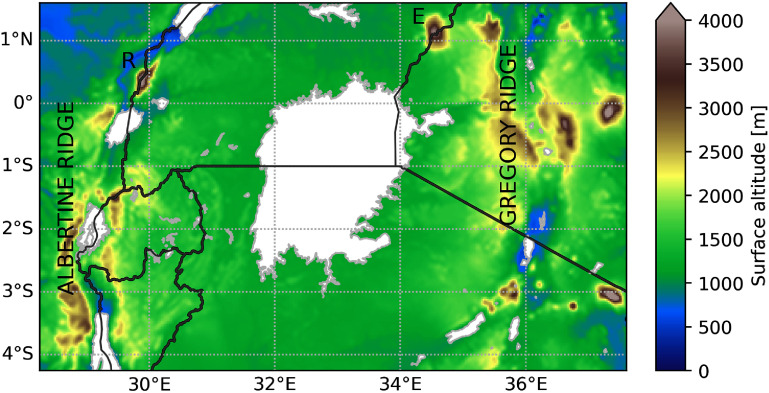
Orographic map of the Lake Victoria region. The Western and Eastern branches of the East-African rift, are indicated as Albertine and Gregory ridge, respectively. The Rwenzori Mountains (*R*) and Mount Elgon (*E*) are indicated. The evaluation domain from Van de Walle *et al* (2021) used in this study is shown which excludes 30 grid points at each side of the full model domain.

The climate model data produced during the investigation conducted by Van de Walle *et al* (2021) serves as the foundation for the analysis in this study. Climate runs were performed with the three-dimensional non-hydrostatic COSMO-CLM version 5.0 (Rockel *et al* 2008). The evaluation domain of Van de Walle *et al* (2021) is used, which excludes 30 grid points at each side of the domain to account for the lateral relaxation zone. The domain covers the Lake Victoria basin and the surrounding western and eastern mountain ranges (figure 1). The model has a horizontal resolution of 0.025^∘^ (∼2.8 km) and has been evaluated for precipitation, lake surface temperature and top-of-atmosphere radiation (Van de Walle *et al* 2020, van Lipzig *et al* 2023). This model has been used to study extreme precipitation and its future intensification around Lake Victoria (Van de Walle *et al* 2021).

The ERA5 reanalysis data (Hersbach *et al* 2020) were used to drive the model simulations for the historical period (2005–2016) from the lateral boundaries (∼31 km). For the future period (2080–2091), the pseudo global warming methodology used in the simulations of (Van de Walle *et al* 2021) follows the procedure as described by Kröner *et al* (2017) and Brogli *et al* (2019) were used. The perturbation fields are calculated from the difference between 30 year historical and future periods from the ensemble mean of the Coupled Model Intercomparison Project Phase 6 (CMIP6, Eyring *et al* 2016, 2019, Almazroui *et al* 2020) for the Shared Socioeconomic Pathway 5-8.5 (SSP5-8.5). The historical period covers the period 1995–2025, which symmetrically surrounds the 10 year run with COSMO-CLM and for the future the period 2070–2100 was chosen. The perturbations fields are calculated for each grid box and each day of the year to provide a climatological annual cycle.

The perturbation fields need to be processed to obtain temporally and spatially smoothed signal, and subsequently interpolated to the ERA5 model levels at an hourly resolution. The temporally smoothening is done by performing a Fourier analysis keeping only three dominant components of the annual cycle (Bosshard *et al* 2011, Brogli *et al* 2019). Spatial smoothening is needed to minimise abrupt transitions between neighbouring grid cells and was done using a Gaussian filter. A linear interpolation was used both for the vertical interpolation to ERA5 model levels as well as for the interpolation from daily to hourly timestamps. The convection-permitting simulations for the future use these processed perturbation fields as initial and boundary conditions.

### 2.2. Model evaluation and observations

The model’s performance was evaluated using Trans-African Hydro-Meteorological Observatory (TAHMO) ground observation datasets providing hourly data for air temperature, relative and specific humidity at 2 m above ground level. Only TAHMO weather stations with at least 6 months of data coverage during the model’s time period, and with observations available for more than 50$\%$ of the time, were used for evaluation. The assessment utilised the root mean square error , mean bias, mean absolute error, and Perkins’ skill score (Perkins *et al* 2007) as statistical metrics. A detailed overview of the evaluation method and data is provided in appendix A.

### 2.3. Population data

We used population data at 0.008^∘^ (∼1 km) spatial resolution from Boke-Olén *et al* (2017). This dataset incorporates a combination of RCP and SSP scenarios and is available for both time periods used in the climate model. Additionally, Boke-Olén *et al* (2017) place a particular focus on the African continent in their study. The dataset builds upon the WorldPop data as a baseline for forecasting future demographic trends. Boke-Olén *et al* (2017) couple RCP and SSP scenarios into their projections to account for urbanisation trends based on RCP scenarios and population projections based on SSP scenarios. In line with the climate model, discussed in section 2.1, we use the RCP8.5-SSP5 scenario. Data extraction is performed for 2010 and 2085, strategically situated midway within both climate model datasets ensuring a balanced representation across the temporal spectrum. We re gridded the population datasets to the resolution of climate model, i.e. from $0.008^\circ \times 0.008^\circ$ to $0.025^\circ \times 0.025^\circ$, using an integral preserving method to conserve the total population across the domain. A sensitivity analysis is performed for other future population projections following the same approach.

Population-weighted temperatures and heat metrics are calculated by multiplying the population of each grid cell by the respective temperature or heat metric in that cell. These products are then added together, and the resulting sum is divided by the total population in the region. This approach ensures that temperatures and heat metrics are appropriately weighted based on the population distribution across the region.

### 2.4. Heat stress metrics

In the process of selecting heat stress metrics, our focus was on commonly employed metrics within the field of heat-health studies (Simpson *et al* 2023). Metrics that take into account solar radiation were deliberately excluded, primarily because assessing surface radiation is challenging due to data scarcity, and the climate change signal for radiation remains uncertain (Bartók *et al* 2017). Furthermore, we limited our scope to metrics reliant on meteorological variables. In this paper, we focus on the humidex index (Masterton and Richardson 1979) (hereafter referred to as ‘humidex’, abbreviated as HD) and the heat index (hereafter abbreviated as HI). These indices are considered appropriate for the African region due to their biophysical basis and general applicability across different climate regions. An overview of their comfort categories is given in table 1.

**Table 1.** Category definition for Heat index NOAA (2014) and Humidex Masterton and Richardson (1979).

Heat index		Humidex	
27 ^∘^C–32 ^∘^C	Caution	20 ^∘^C–29 ^∘^C	Little to no discomfort
32 ^∘^C–41 ^∘^C	Extreme caution	30 ^∘^C–39 ^∘^C	Some discomfort
41 ^∘^C–54 ^∘^C	Danger	40 ^∘^C–45 ^∘^C	Great discomfort
$\gt$54 ^∘^C	Extreme danger	$\gt$45^∘^C	Dangerous

Both humidex and heat index depend on temperature and humidity and are expressed in ^∘^C. However, it is important to note that they do not yield identical results for similar temperature and humidity values (see figure B1 in appendix B). In this study, both are evaluated on an hourly basis. The formulas used can be found in appendix B.

Given our interest in health-related risks, our primary focus is on extreme categories. Heat index values between 41 ^∘^C and 54 ^∘^C are categorised as ‘dangerous’ and values above 54 ^∘^C as ‘extremely dangerous’ (NOAA 2014). Humidex values above 45^∘^C are considered as ‘dangerous’ (Masterton and Richardson 1979). Daily maximum values for both indices are considered. Here we focus on peak heat exposure, which relates to maximum heat stress people experience during the day which is key for physiological stress which is reflected in generic heat stress metrics like the heat index and humidex. As the model is found to be overall warmer and drier, a sensitivity analysis is performed increasing the thresholds for dangerous heat stress for both indices with 1 ^∘^C and 2 ^∘^C.

Since our study incorporates both climate change and future population dynamics, we conduct an analysis to decompose the attribution of each factor to the population exposed to dangerous heat stress. In line with Jones *et al* (2015) and Asefi-Najafabady *et al* (2018), we performed three calculations. The first, known as the ‘climate effect,’ maintains a constant population (i.e. 2010 population) while allowing for changes in the climate (results of the 2080–2091 run compared to the 2005–2016 run period). The second calculation, referred to as the ‘population effect,’ keeps climate conditions constant (i.e. 2005–2016 period) while permitting population changes (2085 compared to 2010 population). In certain regions, both climate and population are expected to increase concurrently. To account for these simultaneous changes, we conducted a third calculation, termed the ‘interaction effect’. This calculation involved subtracting the combined contributions from the climate effect and the population effect from the overall modelled exposure.

## 3. Results

### 3.1. Model evaluation

The regional climate model performs reasonably well in simulating near-surface temperature and humidity across the Lake Victoria basin, though it exhibits a consistent warm (between 0.6 ^∘^C and 1.9 ^∘^C) and dry (between −2.6$\%$ and −18.0$\%$) bias. The model effectively captures the diurnal cycle of temperature, specific humidity, and relative humidity. However, night-time temperatures are generally overestimated, which contributes to a dry bias in night-time relative humidity. During the day, the relative humidity bias may be influenced by a combination of factors, including underestimations of specific humidity at inland station locations and overestimation of temperature in mountainous sites. These tendencies are also reflected in the upper extremes, with the 95th percentile of temperature consistently overestimated and corresponding humidity values underestimated. Detailed statistics are provided in table A2, and diurnal patterns are illustrated in figures A1–A3.

Although the warm bias increases the heat stress, this effect is partly offset by the dry bias as the heat stress metrics used in this study are sensitive to both temperature and humidity. In the upper extremes, a decrease in relative humidity of about 4% will equally reduce the heat stress as a temperature decrease of about a 1 ^∘^C (based on the gradient of the isopleths shown in figure B1). However, this compensating effect relies on the assumption that the warm and dry biases occur simultaneously, which is not always the case. When they do not align, the warm bias alone may lead to an overestimation of heat stress severity, with a magnitude comparable to the temperature bias itself.

### 3.2. Population change

**Figure 2.**
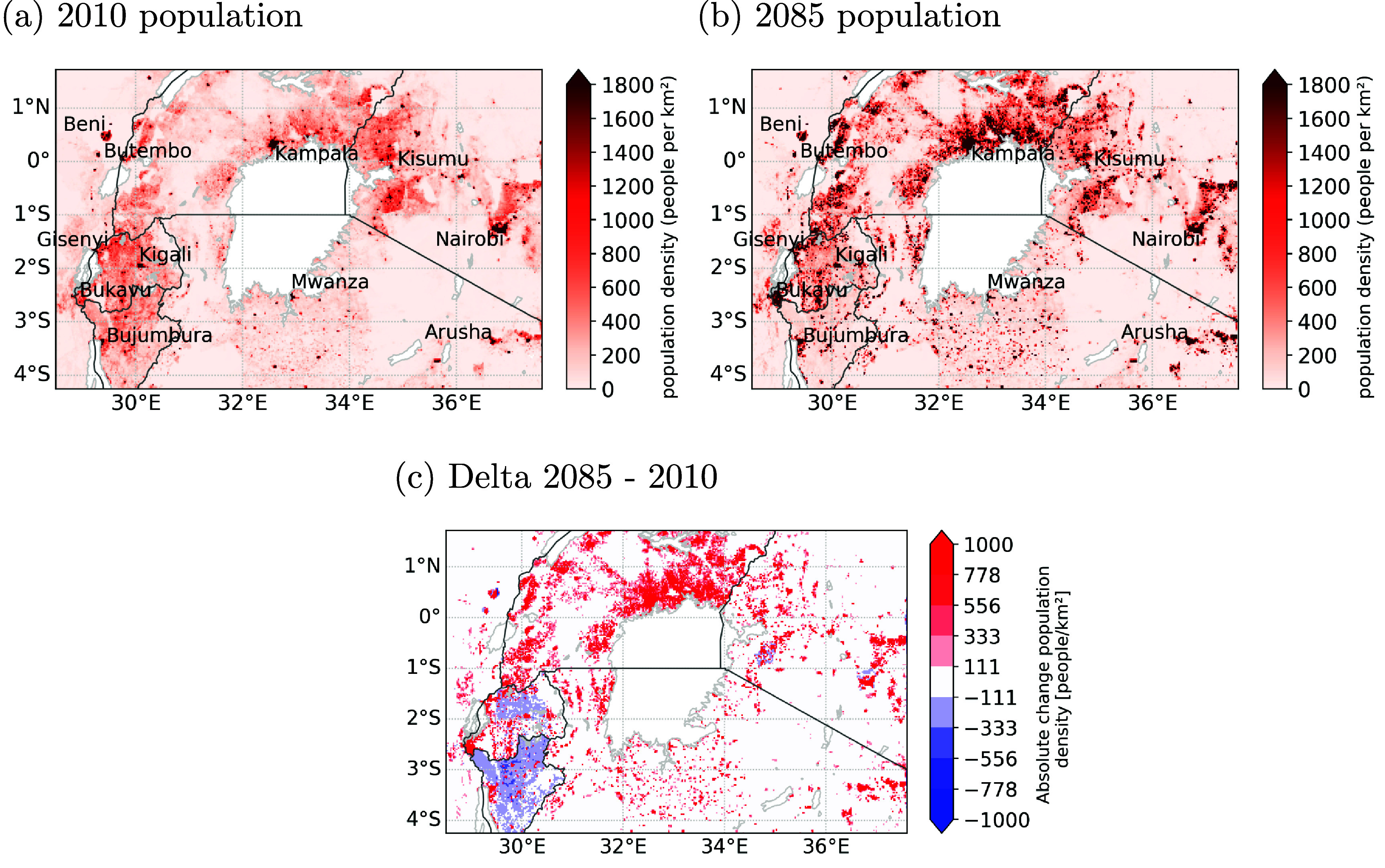
Population density per km^2^ in (a) the year 2010, (b) projected in the year 2085 under RCP 8.5-SSP5, both according to Boke-Olén *et al* (2017), and (c) change between 2010 and 2085.

This section presents the re gridded population data from Boke-Olén *et al* (2017). Across the entire region, the total population is projected to increase from approximately ∼101.1 million in 2010 to approximately ∼278.8 million in 2085 under a RCP8.5-SSP5 scenario. Specifically, the northern shore of Lake Victoria, running from the Rwenzori Moutains in Rwanda to Mount Elgon in Kenya experience a substantial increase in population density, as depicted in figure 2. This trend is also projected for cities such as Nairobi and Kisumu in Kenya, Kampala in Uganda, Kigali and Gisenyi in Rwanda, Butembo, Beni and Bukavu in the Democratic Republic of Congo, Bujumbura in Burundi and Mwanza and Arusha in Tanzania. Conversely, the projected population for Burundi and Rwanda shows an overall decrease across the country, with some exceptions in certain cities like Bujumbura.

### 3.3. Climate change

Based on the climate model data from Van de Walle *et al* (2021), there is a noticeable and consistent upward trend projected in the daily mean and maximum temperatures throughout the region, resulting in prolonged periods of exposure to extreme heat. Figures C1 and C2 in appendix C give an overview of the distributions of the temperature and relative humidity values in the 2005–2016 and 2080–2091 climate model run. Figure C3 in appendix C provides the average hourly temperature variations throughout the region, including the 95th and 99th percentile values. Changes in mesoscale affect temperature and are driven by the varying thermal contrast between land and lake, as land warms faster than the lake (a 3.5 K domain-averaged increase in near-surface temperature versus a 2.4 K increase over the lake) (Van de Walle *et al* 2021). In particular, at night, this thermal contrast weakens amplified by a stronger nighttime warming (4.2 K compared to 3.0 K during the day), reducing the mesoscale circulation from land to lake (lake breeze). Opposite, during the day, the circulation from lake to land increases. This pattern influences cloud formation and thunderstorms, which are significant for radiation and extreme heat. Furthermore, the CMIP6 ensemble mean projects a moderate reduction in easterly winds in the future, influenced by orography and land-lake contrast (Van de Walle *et al* 2021).

The daily mean temperature across the domain is projected to increase from 22.4 ^∘^C to up to 26.1 ^∘^C (+3.7 ^∘^C) in future. The population-weighted daily mean temperature (using the 2010 population) is projected to increase from 21.5 ^∘^C in 2005–2016 climate run to up to 25.3 ^∘^C (+3.8 ^∘^C) in the 2080–2091 climate run. The daily maximum temperature and population-weighted daily maximum temperature increase from 29.0 ^∘^C to up to 32.3 ^∘^C (+3.3 ^∘^C) and from 28.4 ^∘^C to up to 31.7 ^∘^C (+3.3 ^∘^C), respectively.

**Figure 3.**
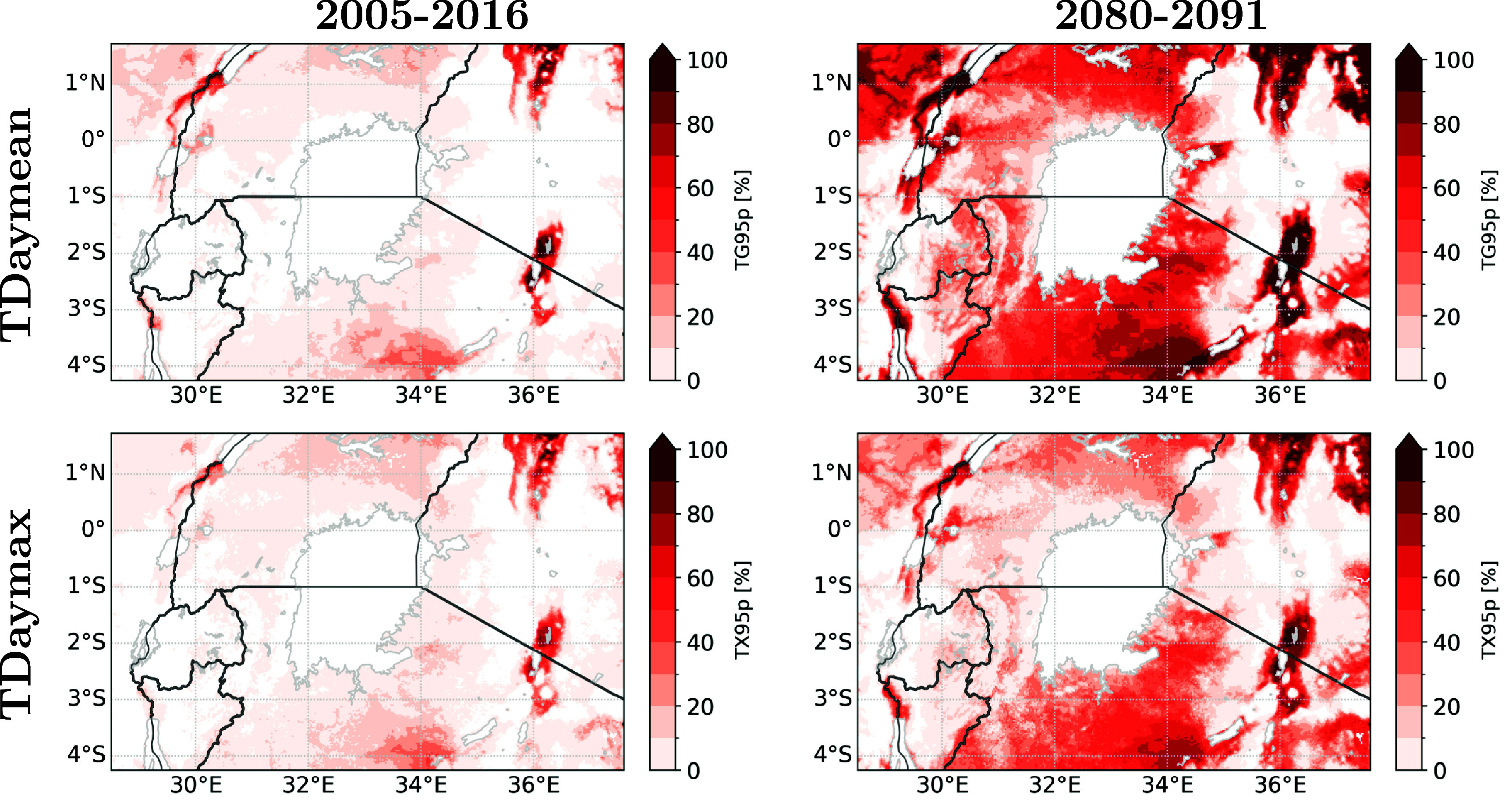
Percentage of days exceeding the 95th percentile of the daily mean temperatures across the domain (abbreviated as $TG_{95p}$, threshold is 27.7 ^∘^C, top row) and daily max temperatures across the domain (abbreviated as $TX_{95p}$, threshold is 37.1 ^∘^C, bottom row). Percentile thresholds are calculated over the baseline period 2005–2016.

More extreme warm days (quantified by the 95th percentile of daily mean and maximum temperatures across the domain excluding lake pixels) are found in the valleys, illustrated in figure 3, indicating a prominent upward trend in temperature extremes. Additionally, the southern shore of Lake Victoria is characterised by more extreme hot days compared to the northern part. However, these identified regions do not necessarily coincide with the areas where people reside, as depicted in figure 2.

The population-weighted exceedance days for the 95th percentile of the daily mean temperature (i.e. 27.7 ^∘^C) is expect to rise from 7.8 days per year in the 2005–2016 climate run to up to approximately three months per year in the 2080–2091 climate run. For daily maximum temperatures (i.e. 37.1 ^∘^C), a similar count of exceedance days in the 2005–2016 climate run (i.e. 9.0 days per year) escalates to up to 1.5 months per year in the 2080–2091 climate run.

### 3.4. Change in heat stress metric

**Figure 4.**
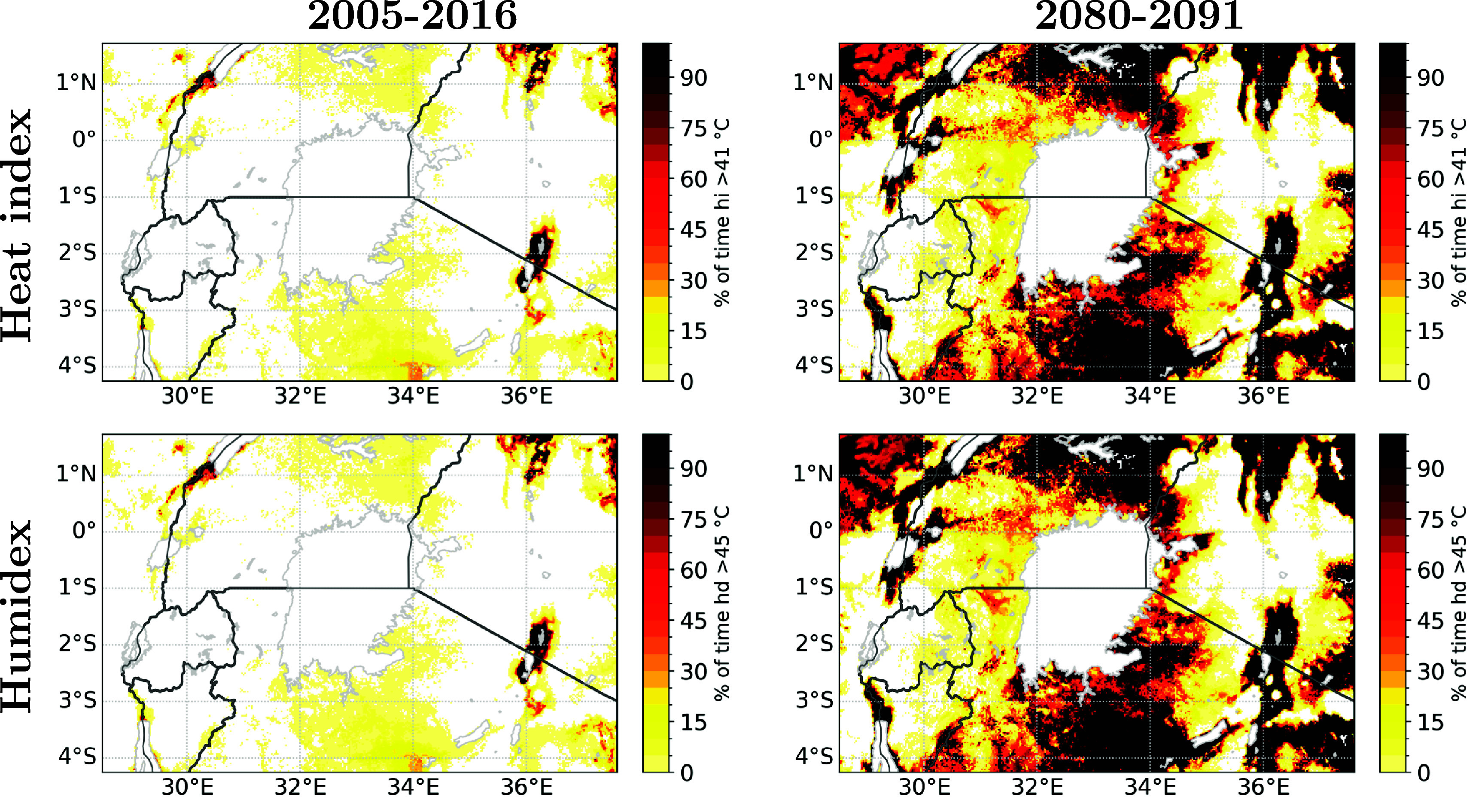
Percentage of days during a year within the Danger and Extreme danger category for Heat index (top row) and Dangerous category for Humidex (bottom row) for the 2005–2016 (left column) and 2080–2091 (right column) climate context.

To address the climate induced change in heat stress, the percentage of days considered dangerous for both the heat index and humidex was evaluated (figure 4). In the 2005–2016 climate run, the dangerous threshold is only exceeded for a limited amount of time (i.e. 0.7 d yr^−1^ for HI and 0.8 days per year for HD). Moreover, exceedance predominantly manifests in sparsely populated regions. In the future, both metrics show a sharp increase, with nearly all days exceeding the threshold in low-altitude, sparsely populated areas. For the dangerous categories, the population-weighted exceedance is projected to rise to up to 36.0 days per year for heat index and up to 41.2 days per year for humidex in future.

### 3.5. Change in heat exposure

**Figure 5.**
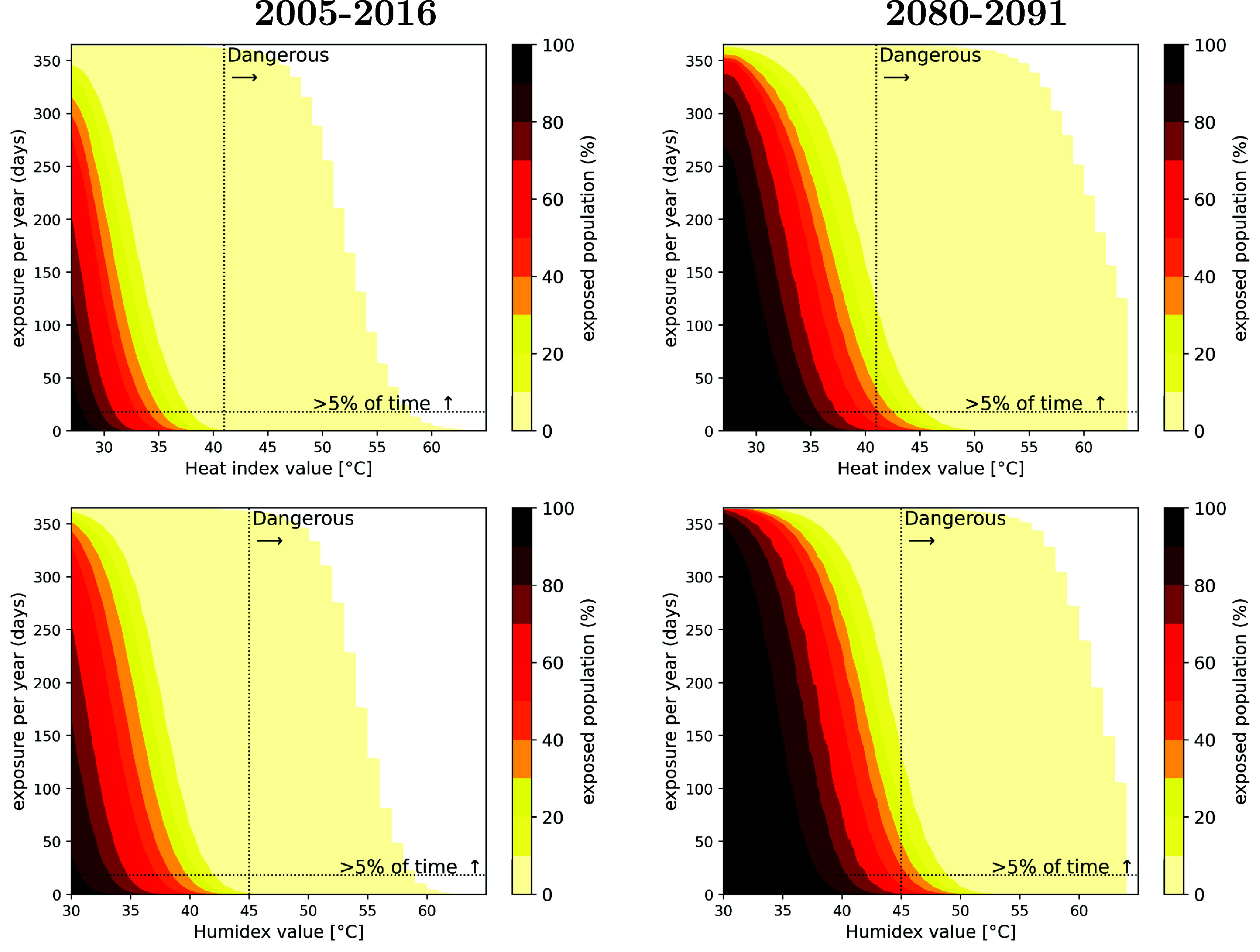
Share of population ($\%$) exposed to certain threshold of heat stress (heat index on top row, humidex on bottom row) for a certain number of days per year. From left to right: 2005–2016 climate run and 2010 population, 2080–2091 climate run and 2085 population. The vertical and horizontal line indicate the threshold for the heat stress metric and 5$\%$ time exceedance (i.e. ∼18 days per year).

Using this modelling methodology, in 2010, 1 million people (i.e. 0.99$\%$ of the study population) were exposed to dangerous heat stress for more than 5$\%$ of the days (figure 5). In future, this is projected to increase to up to 121.7 million people or around 44$\%$ of the future population. For 0.2 million people (0.2$\%$ of the population) today and up to 76.9 million in future (27.6$\%$ of the population) dangerous heat stress is expected to be reached for more than 15$\%$ of days (i.e. ∼55 d yr^−1^, see figure D1 in appendix D).

**Figure 6.**
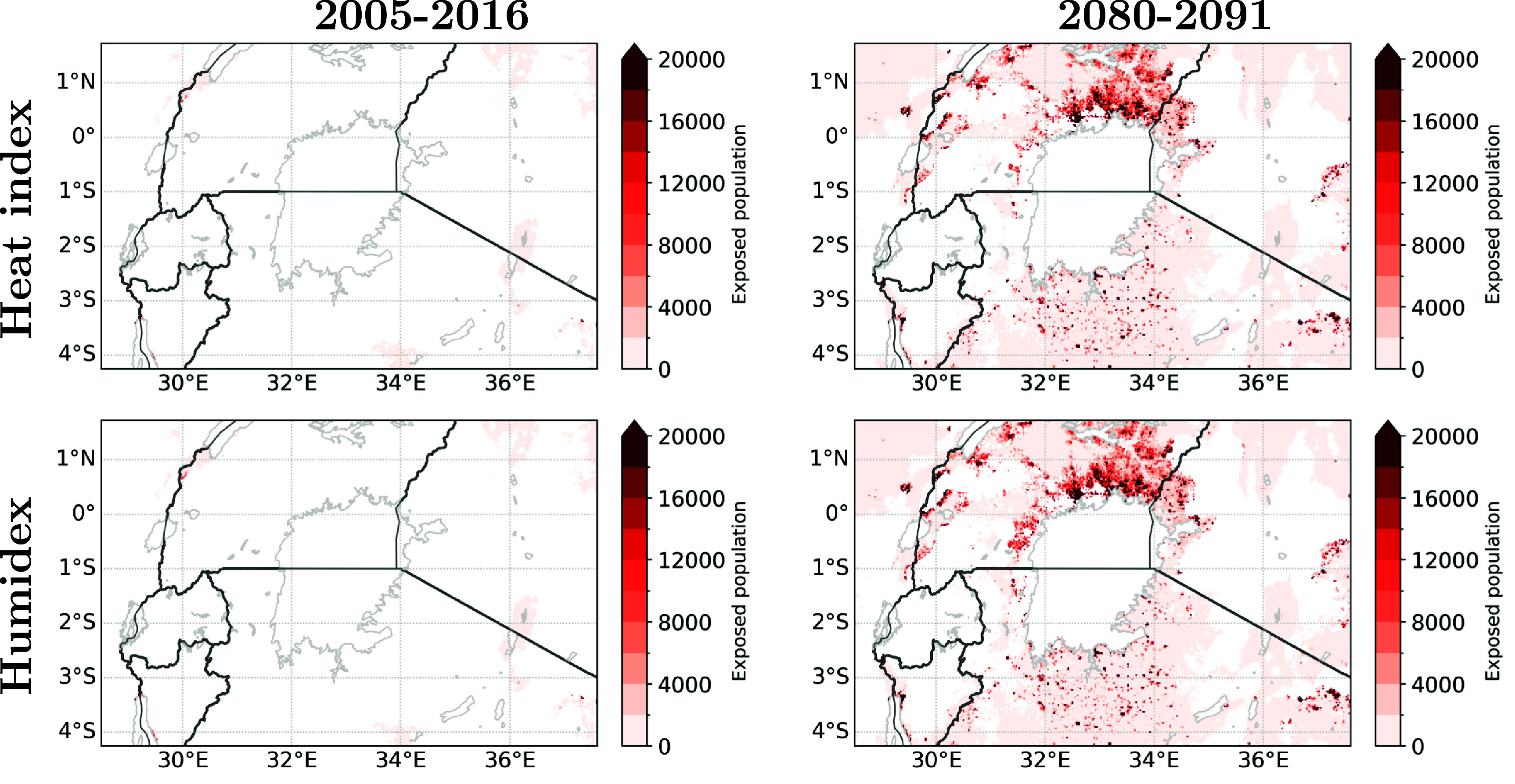
Population exposed to the dangerous threshold of heat stress (heat index on top row, humidex on bottom row) during more than 5$\%$ of days per year. From left to right: 2005–2016 climate and 2010 population, 2080–2091 climate and 2085 population.

It is crucial to identify the regions that are susceptible to dangerous heat stress. Figure 6 highlights the areas with high numbers of inhabitants exposed to dangerous heat stress for more than 5$\%$ of the year (i.e. ∼18 days). The northern shore of Lake Victoria emerges as one such region, including the city of Kampala. The southern shore of Lake Victoria and the southeastern shore of the Albert lake, are two other regions with high exposure, though they show a more scattered pattern in line with the more scattered picture we find for the future population scenario in this region (shown in figure 2(b)). Further areas where cities such as Kisumu, Nairobi, Arusha, Bujumbura, Beni, Rutshuru are located also appear. Although the eastern shore of Lake Kivu show regions with a high future population density, this area does not appear as a highly exposed region which is likely linked to the higher altitudes characterising this region.

### 3.6. Attribution of climate and population to changes in heat exposure

**Figure 7.**
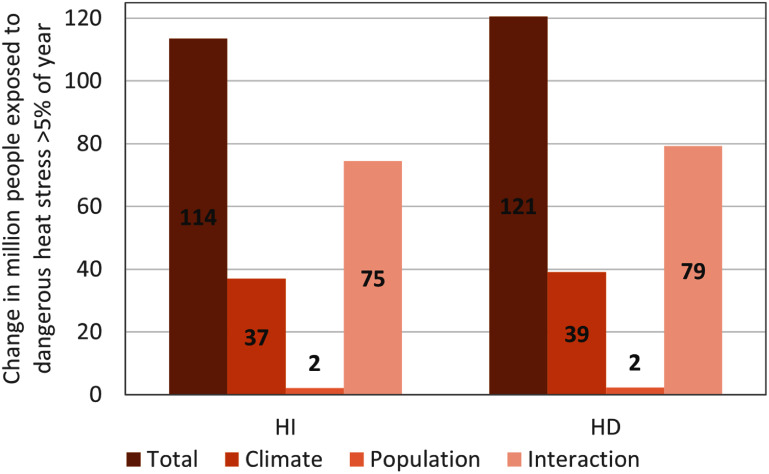
Decomposition of climate, population and interaction attribution to total people (in million) exposed to dangerous heat stress for more than 5$\%$ days per year (i.e. ∼18 days) for heat index (left) and humidex (right).

Across the region, up to 113.6/120.6 million more people are projected to be exposed to dangerous heat stress by 2085 based on the heat index/humidex respectively (figure 7). About 33$\%$ of this additional exposure for inhabitants can be attributed to the impact of climate change, while the impact of population change attributes for about 2$\%$. The biggest share (i.e. 66$\%$) of the increase can be linked to the ‘interaction effect’, referring to areas where temperatures are getting more extreme and population is increasing.

Across the study area the proportional changes between the climate, population and interaction attribution differs as illustrated in figure D2 in appendix D. The population effect is limited and appears in some lower altitudes in the region. The reason for this is that in most areas the exposure to dangerous heat in the 2005–2016 climate context is present for less than 5$\%$ of the time (i.e. ∼18 days), meaning that a rise in population has only a marginal impact on altering the heat exposure. For the biggest part of the region, the climate effect is the main driver where it has a contribution of ∼95$\%$, complemented with ∼5$\%$ contribution for the interaction effect. For the northern shore of Lake Victoria, the pattern is more scattered. The areas with lower population densities (shown in figure 2(b)) have a contribution of climate change ranging around 75$\%$–80$\%$, while for the areas with higher population densities this ranges between 10$\%$ and 55$\%$. The interaction effect complements the climate effect, reflecting both rising heat exposure and significant population growth. The lower share for the climate effect and higher one for the interaction effect in the higher populated areas in the northern shore, also appears for areas with higher population densities on the southern shore and some areas where cities as Arusha, Bujumbura, Nairobi, Kisumu, Bukavu and Beni are located.

## 4. Discussion

Our study primarily focused on assessing the impact of climate change (SSP5-8.5 scenario) and population change (RCP8.5-SSP5) on dangerous heat stress exposure within the vicinity of Lake Victoria, employing a resolution of 0.025^∘^ (∼2.8 km). The global trends and patterns found in our study are in line with the research of Asefi-Najafabady *et al* (2018). An increase in heat exposure more than twice as high for Uganda compared to other countries in the Greater Lakes Region was highlighted. Although our study domain only covers the southern part of Uganda, it is evident that this region experiences a substantially higher population exposed to dangerous heat stress. Similar to our study, both climate change and the interaction between climate change and population change were found to be major contributors to this increase. However, it is worth noting that Asefi-Najafabady *et al* (2018) reported higher contributions from the population effect for Uganda, Tanzania, and Kenya, but this could be attributed to differences in the specific regions covered by our study. Our study goes further by using a finer spatial resolution, which allows us to capture small-scale variability more precisely. This finer resolution enables us to identify and analyse the finer spatial correlation between population and heat exposure, providing a more detailed understanding of the localised impacts of heat stress. Even though the added value of CPM models is apparent from the spatial variability of percentage during a year with extreme heat stress (figure 4), a more in-depth identification of the added value of CPM in dry and humid heat stress would be an interesting topic for future research.

In tropical Africa, so-called humid heat stress, which occurs when the human body is exposed to a combination of high temperatures and high humidity, is frequently occurring. Recent research has indicated that entrainment of dry air in the air between the surface and 3 m height, limiting deep convection, leads to high near-surface humid heat stress (Duan *et al* 2024). The use of such CPM models, like we do here, to study tropical heat stress is thought to be beneficial compared to models with parametrised convection. Moreover CPMs better represent the diurnal cycle of clouds, which is crucial for accurately assessing extreme daily temperatures and moisture, and therefore, for heat stress metrics (Brisson *et al* 2016, van Lipzig *et al* 2023). These are all sound reasons to use CPMs for studies on heat stress, but an in depth analysis of heat stress in CPMs compared to models that parametrise convection is a topic for future research.

In our analysis, we employed the ensemble mean for a high-end climate change scenario (SSP5-8.5), acknowledging that it may not be the most realistic scenario Hausfather and Peters (2020). Nevertheless, it remains relevant in the context of robust adaptation measures that holds for the worst possible outcome. Therefore, our estimates are referred to as the upper range of the expected change. Additionally, as discussed in section 2.1, our model is warmer compared to the available observation.

A sensitivity analysis for the threshold values for dangerous heat stress has been elaborated. Increasing the thresholds with 1 ^∘^C decreases the population exposed to dangerous heat stress for more than 5$\%$ of the days from up to 121.7 million people to around 100 million people or around 36$\%$ of the future population. Increasing the thresholds with 2 ^∘^C decreases the exposed population further to around 71 million people or around 26$\%$ of the future projected population.

Next to the RCP8.5-SSP5 population scenario, a sensitivity analysis is carried out for different population projections. Population projections highly vary across the different scenarios ranging between around 275–280 million people for the SSP5 and SSP1 scenarios and around 590 to up to 683 million for the SSP3 en SSP4 scenarios in 2085. The SSP3 and SSP4 scenarios project significant population growth in this region, generally assuming high population growth in developing countries and low to medium migration rates (Samir and Lutz 2017). In contrast, SSP1 and SSP5 project lower population growth rates in this region, driven by investment in education and health globally. SSP2 is considered the middle of the road scenario and projects a population around 410 million in the region in 2085. In the SSP4-RCP8.5 scenario, the population exposed to dangerous heat stress for more than 5$\%$ of the days is projected to up to 304 million people (or around 44$\%$ of the projected population for that scenario). Decreasing the heat stress thresholds with 2 ^∘^C decreases this to around 179 million people or around 26$\%$. These relative numbers are in line with the main population scenario chosen for this study.

Another noteworthy limitation is that the climate model used did not incorporate an urban scheme. For instance, Brousse *et al* (2020) modelled a notably stronger urban heat island intensity of up to 3 ^∘^C on average above the more densely urbanised zones in Kampala during night-time compared to former urbanisation extent of the beginning of the 21st century. However, for daytime, where we are focusing on here, differences are smaller. Implementing an urban scheme in convection-permitting regional climate models would support improved heat stress assessments in urban areas Wouters *et al* (2016). Additionally, the impact of changing anthropogenic heat due to varying population density was not addressed, which would be of interest in addition to the implementation of the urban scheme.

The comparison of two heat metrics revealed agreement in the categories of dangerous heat stress, aligning with the expected treatment of temperature and humidity as crucial determinants of heat stress (see figure B1 in appendix B). However, variations in time estimated in each heat stress category were observed for the lower categories (see figure D3), as well as how these variations manifest across the study region (see figures D4 and D5). Additionally, due to the heterogeneity of population density within the region, deviations in the population-weighted time in each heat stress category were observed for the lower categories. For example, in future, population-weighted, 56.2$\%$ of the time is considered to fall within the category ‘Extreme caution’ for the heat index, while 34.4$\%$ of the time is categorised under ‘Great discomfort’ for the humidex. Both categories are the one beneath the ‘Dangerous’ categories in both indices. These discrepancies highlight the sensitivity of heat metrics to the choice of specific variables and thresholds. Consequently, the selection of a particular metric can influence the conclusions drawn from the analysis (Simpson *et al* 2023). Caution is recommended when interpreting and generalising the findings, taking into account the potential variability introduced by different metrics.

The implications of the study’s findings for the region are multifaceted. The findings of this study concur with Miranda *et al* (2023) that countries in the region, including Uganda and Democratic Republic of Congo are among the most vulnerable countries in Africa to extreme temperatures. This implies that these countries will have higher cooling requirements to adapt to the heat stress. Impact of exposure to heat stress on health were found in East Africa, where there is a significant increase in the relative risk of mortality for children under five years old during high heat exposure (Brimicombe *et al* 2024). In Nairobi’s informal settlements, increasing temperatures were found to significantly associate with mortality in children and deaths caused by chronic, long-lasting conditions (not transmitted through infection) (Egondi *et al* 2012). In addition, the region is dependent on agriculture which is quite sensitive to heat stress and may affect the food production. In an exploration of the wet bulb global temperature study by Yengoh and Ardö (2020) on small holder farmers, it becomes evident that the countries in the region are currently grappling with the challenges of heat stress. Tanzania and Kenya stand out as the most severely affected when compared to Uganda. Effects of heat stress in Tanzania were found to reduce total labour, though only in the male population (Lee *et al* 2021). The majority of communities in East Africa exhibit common characteristics such as poverty, strong dependence on agriculture and outdoor activities, residence in densely populated neighbourhoods, tin-walled housing infrastructure, and a lack of sufficient vegetation. These conditions collectively render them particularly susceptible to the adverse effects of heat stress. Even with the available evidence on the projected extreme temperatures, most of the adaptation plans in place especially by humanitarian agencies focus on floods and droughts (Anticipation Hub 2023). As mentioned before, the whole region is characterised by a rapid population increase and urbanisation (Liu *et al* 2017, Asefi-Najafabady *et al* 2018, United Nations 2022). Unfortunately the provision services and infrastructure do not match the urbanisation rate thus creating more slums that are vulnerable to climate risks including heat. These informal settlements often experience higher temperatures compared to other parts of the city (Scott *et al* 2017, Van de Walle *et al* 2022). Scott *et al* (2017) recommend better urban planning to address heat stress especially in these slum areas. Sseviiri *et al* (2022) propose similar strategies to address heat stress including greening the city, promotion of non-motorised transport corridors and tree planting.

Finally, our study only reflects on the expected local changes in exposure to dangerous levels of heat stress for the local populations. Nevertheless, as mentioned above other important health risks are expected to be impacted by regional climate changes like exposure to vector-borne diseases (Brousse *et al* 2019, Morlighem *et al* 2022), flooding and water availability (Lwasa 2010), or food security (Lwasa *et al* 2014), amongst others (Serdeczny *et al* 2017). Future studies should therefore pay particular attention to the complexities underpinning adaptive and mitigative solutions.

## 5. Conclusion

### 5.1. Main findings

This study underscores the substantial impact of climate change on extreme heat stress in the Lake Victoria region and the consequent population exposure. The results suggest that, under the combined influence of population increase and climate change, consistently modelled with an SSP5-RCP8.5 scenario, the duration of dangerous heat stress could escalate from less than one day per year in 2010 to exceeding one month per year by 2085. This increase poses a substantial risk for up to 122 million people, which equates to approximately ∼44$\%$ of the future population in the region. These individuals may face dangerous heat stress for more than 5$\%$ of the time (i.e. ∼18 days) on an annual basis. Moreover, up to 28$\%$ of the future population could experience dangerous heat stress for more than 15$\%$ of the time (i.e. ∼55 days). About 33$\%$ of this increased population exposure can be attributed to the impact of climate change, while the impact of population change attributes for about 2$\%$. The majority of this rise, constituting 66$\%$, is attributed to the ‘interaction effect,’ emphasising the critical interplay between temperature extremes and population growth. The regions with the highest concentration of inhabitants exposed to dangerous heat stress are primarily situated along the northern shore of Lake Victoria and the region south of it, including their cities.

Our study highlights the pressing relevance of a future increase in heat stress exposure in highly vulnerable countries such as Uganda, Tanzania, Kenya, and the Democratic Republic of Congo. Socioeconomic factors like poverty, dense informal settlements, and poor housing, amplifies the impacts of rising temperatures on health, labour and agriculture. Current adaptation efforts by humanitarian agencies focus largely on floods and droughts. Our results can be applied to develop heat stress adaptation strategies for the region. Moreover, our work underlines the need to avoid the worst-case heat exposure by cutting greenhouse gas emissions.

### 5.2. Limitations

While this study provides valuable insights, several limitations must be acknowledged. Despite the valuable insights provided, several limitations must be acknowledged. Climate models, including COSMO-CLM, are primarily calibrated for mid-latitude regions and may exhibit larger biases in East Africa. Specifically, the model tends to simulate warmer (between 0.6 ^∘^C and 1.9 ^∘^C) and drier (between −2.6% and −18.0%) conditions compared to observations.These discrepancies highlight the need for improved representation of cloud processes and humidity in regional climate modelling. Additionally, the current simulations do not account for urban heat island effects, which are critical for assessing heat stress in densely populated cities. Lastly, the exclusive use of the SSP5-RCP8.5 scenario, while useful for exploring extreme outcomes, may not reflect the most probable future trajectory.

### 5.3. Future work

Future research should aim to reduce model biases through improved parametrisations tailored to tropical climates, particularly regarding cloud-radiation interactions and moisture dynamics. Incorporating urban schemes into regional climate models would enhance the accuracy of heat stress projections in urban areas. To broaden the policy relevance of the findings, future studies should also explore a range of socioeconomic and emissions scenarios, including lower-emission pathways. Integrating the global warming level framework would further align the results with international climate targets and adaptation planning.

## Data Availability

The data cannot be made publicly available upon publication because the cost of preparing, depositing and hosting the data would be prohibitive within the terms of this research project. The data that support the findings of this study are available upon reasonable request from the corresponding author, DR.
